# Exploring the Influence of Recreational Cannabis Legalization on Women’s Perceptions and Experiences with Perinatal Cannabis Use: A Qualitative Meta-synthesis

**DOI:** 10.1007/s10995-026-04228-5

**Published:** 2026-02-25

**Authors:** Kassandra Maturino, Jamie Morton, Karry Weston, Allison Anbari

**Affiliations:** Sinclair School of Nursing, University of Missouri, Columbia, MO USA

**Keywords:** Cannabis, Pregnancy, Breastfeeding, Public policy, Qualitative review

## Abstract

**Objective:**

We aimed to explore how women living in places with legalized recreational cannabis perceive perinatal cannabis use, and how legalization potentially impacts their experiences.

**Methods:**

Between September 2024 and December 2025, we searched databases including PubMed, CINAHL, Scopus, PsycINFO, and Web of Science. All research reports were screened and evaluated based on the inclusion and exclusion criteria. Theory-generating qualitative meta-synthesis methodology was used to extract, analyze, and synthesize the findings from included qualitative research reports. Quality appraisal of each study was also conducted.

**Results:**

Nineteen qualitative research reports were included, and eight themes were identified. Themes included (1) recreational cannabis legalization, (2) clinical policies and practice, (3) stigma and discrimination, (4) access to and desire for information, (5) clinician-patient relationships, (6) most trusted sources: family and friends, (7) perceptions and experiences and (8) self-management.

**Conclusion:**

This qualitative meta-synthesis highlights the complex interplay of public policy, institutional and community, interpersonal, and intrapersonal factors influencing women’s perceptions of and experiences with perinatal cannabis use, as well as provides valuable insight into the decision-making process. These implications can help inform targeted public health and clinical practice interventions to address the complexities of cannabis use during the perinatal period.

## Significance

Recreational cannabis legalization is rapidly expanding, and cannabis use during the perinatal period could increase even further as a result. Despite known adverse effects on maternal and child health, the growing literature suggests women continue to use cannabis perinatally for its perceived symptom management benefits and safety misconceptions. Social, structural, and contextual factors influence women’s decision-making about perinatal cannabis use. Although this is a multi-faceted issue, the lack of clear cannabis messaging, perinatal health and cannabis education, and non-stigmatizing patient-centered healthcare for the perinatal population are critical gaps. Our theoretical model visually illustrates the intersecting factors driving perinatal cannabis use decisions.

## Cannabis Legalization

Nine countries have legalized recreational cannabis use nationwide, while laws in the United States (US) and Australia vary by state or jurisdiction (Venditti, 2024). In the US, 24 states, two territories, and the District of Columbia have legalized recreational cannabis for adult use (DISA Global Solutions, 2025). Cannabis legalization benefits include justice decriminalization, drug product regulation, and taxation, yet the safety and health-related impacts on vulnerable populations are poorly understood. As recreational cannabis legalization expands and patterns of maternal cannabis use are documented globally, it is critical to understand the implications for pregnant and parenting women.

### Perinatal Cannabis Use

Perinatal cannabis use is defined as use before, during, or after pregnancy, including during breastfeeding (The American College of Obstetricians and Gynecologists [ACOG], 2017). According to the most recent data on cannabis use during pregnancy by the Substance Abuse and Mental Health Services Administration (2022), 8% (*n* = 164,000) of US pregnant women aged 15 to 44 years reported past month cannabis use in 2020. Prior research has shown that the prevalence of cannabis use during pregnancy is highest among Black, low-income, unmarried, and younger women (Ko et al., 2015, 2020). Although relatively weak associations between maternal cannabis use and adverse infant health outcomes have been estimated, in-utero cannabis exposure has been linked to infant low birth weight (LBW), premature delivery, small for gestational age, and longer neonatal intensive care admissions (Lo et al., 2024). Additionally, recent studies demonstrate an increased risk for attention-deficit/hyperactivity disorder (ADHD) and poor brain neurodevelopment (Bassalov et al., 2024; Paul et al., 2021). While cannabis use during breastfeeding is significantly understudied, two studies found exclusively breastfed infants ingest about 2.5% of the maternal tetrahydrocannabinol (THC) dose used, which may be higher with chronic use and increases in potency (Baker et al., 2018; Bertrand et al., 2018).

Cannabis use among women who are considering pregnancy, pregnant, or breastfeeding is discouraged (Office of the Surgeon General, 2019; ACOG, 2017). Despite these recommendations, pregnant and parenting women continue to use cannabis for its perceived symptom management benefits and misconceptions about legality and safety. Studies have shown that cannabis use during pregnancy frequently occurs in the first trimester for self-management of nausea and vomiting, pain, stress, anxiety, and depression (Vanstone et al., 2022). Limited research suggests that cannabis use during pregnancy is associated with several adverse maternal health outcomes including increased risk for gestational hypertension, preeclampsia, weight issues, and placental abruption (Young-Wolff et al., 2024a).

### Research Aims

Understanding the multi-level factors that influence women’s use of cannabis during the perinatal period can provide valuable information to improve public health interventions and clinical practices. The question guiding our qualitative meta-synthesis was: What do women living in a place with legalized recreational cannabis think about perinatal cannabis use, and how does legalization impact their experiences with perinatal cannabis use?

## Methods

### Design

Theory-generating qualitative meta-synthesis (QMS) methods were used to extract, analyze, and synthesize qualitative research findings from peer-reviewed primary and secondary qualitative research reports (Finfgeld-Connett, 2018). The QMS process extends past combining findings from research reports and moves to the development of a theory that is transferable (i.e., generalizable) beyond the original samples. This broadens the applicability of the newly synthesized theory and its capacity to support decision-making and action initiatives (Finfgeld-Connett, 2018). Ethical review board approval was not needed because the data used was already published and deidentified.

### Search Strategy

Guided by a health sciences librarian, we searched the literature focusing on childbearing-aged women’s (18 − 44 years) perceptions of and experiences with perinatal cannabis use where cannabis is legal for recreational purposes, including any country, state, and/or jurisdiction. Because recreational cannabis was first legalized in Colorado in 2012, only research reports published in 2012 and after were included. Searches were limited to English and completed between September 2024 through December 2025. Multiple databases were searched, including the Cumulative Index to Nursing and Allied Health Literature (CINAHL), PubMed, Scopus, PsycINFO, and Web of Science (Table 1). The PRISMA reporting guidelines provided a structured framework for reporting our search processes (Page et al., 2021). Backward and forward searching of included reports were conducted to identify any other relevant reports (Finfgeld-Connett, 2018). Reports focusing on other substances and outside of the perinatal period were excluded. Reports conducted in places with other forms of cannabis legalization such as medicinal, cannabidiol, and decriminalization only were also excluded (Table 2).

**Table 1:** PubMed search strategy

Search ID	Search terms	Search options or filters	Results
S10	(((cannabis OR marijuana OR THC OR Tetrahydrocannabinol) AND (pregnancy OR perinatal OR prenatal OR antenatal OR postpartum OR intrapartum OR peripartum OR postnatal)) AND (perception OR perceptions OR attitude OR attitudes OR knowledge OR belief OR beliefs)) AND (qualitative OR themes OR thematic OR grounded theory OR ethnographic OR ethnonursing OR phenomenological OR focus groups OR focus group or interview OR interviews)	All Fields	60
S9	((((cannabis OR marijuana OR THC OR Tetrahydrocannabinol) AND (pregnancy OR perinatal OR prenatal OR antenatal OR postpartum OR intrapartum OR peripartum OR postnatal)) AND (legalization OR liberalization OR policy OR policies OR law OR laws)) AND (perception OR perceptions OR attitude OR attitudes OR knowledge OR belief OR beliefs)) AND (qualitative OR themes OR thematic OR grounded theory OR ethnographic OR ethnonursing OR phenomenological OR focus groups OR focus group OR interview OR interviews))	All Fields	20
S8	((((cannabis OR marijuana OR THC OR Tetrahydrocannabinol) AND (pregnancy OR perinatal OR prenatal OR antenatal OR postpartum OR intrapartum OR peripartum OR postnatal)) AND (legalization OR liberalization OR policy OR policies OR law OR laws)) AND (perception OR perceptions OR attitude OR attitudes OR knowledge OR belief OR beliefs)) AND (qualitative OR themes OR thematic OR grounded theory OR ethnographic OR ethnonursing OR phenomenological OR focus groups OR focus group OR interview OR interviews))	Title/Abstract	8
S7	(((cannabis OR marijuana OR THC OR Tetrahydrocannabinol) AND (pregnancy OR perinatal OR prenatal OR antenatal OR postpartum OR intrapartum OR peripartum OR postnatal)) AND (perception OR perceptions OR attitude OR attitudes OR knowledge OR belief OR beliefs)) AND (qualitative OR themes OR thematic OR grounded theory OR ethnographic OR ethnonursing OR phenomenological OR focus groups OR focus group or interview OR interviews)	Title/Abstract	20
S6	(((cannabis OR marijuana OR THC OR Tetrahydrocannabinol) AND (pregnancy OR perinatal OR prenatal OR antenatal OR postpartum OR intrapartum OR peripartum OR postnatal)) AND (perception OR attitude OR knowledge OR belief OR beliefs)) AND (qualitative OR themes OR thematic OR grounded theory OR ethnographic OR ethnonursing OR phenomenological OR focus groups OR focus group or interview OR interviews)	All Fields	54
S5	(((cannabis OR marijuana) AND (pregnancy OR perinatal OR prenatal OR antenatal OR postpartum)) AND (perception OR attitude OR knowledge OR belief OR beliefs)) AND (qualitative OR themes OR thematic OR grounded theory OR ethnographic OR ethnonursing OR phenomenological OR focus groups OR focus group or interview OR interviews)	All Fields	53
S4	(((cannabis OR marijuana) AND (pregnancy OR perinatal OR prenatal OR antenatal OR postpartum)) AND (perception OR attitude OR knowledge OR belief OR beliefs)) AND (qualitative OR themes OR thematic OR grounded theory OR ethnographic OR ethnonursing OR phenomenological OR focus groups OR focus group or interview OR interviews)	All Fields	53
S3	(((cannabis OR marijuana) AND (pregnancy OR perinatal OR prenatal OR antenatal OR postpartum)) AND (perception OR attitude OR knowledge OR belief OR beliefs)) AND (qualitative OR themes OR thematic OR grounded theory OR ethnographic OR ethnonursing OR phenomenological OR focus groups OR focus group or interview OR interviews)	All Fields	52
S2	((((perinatal cannabis use OR prenatal cannabis use OR cannabis use during pregnancy or cannabis use while breastfeeding) AND (women of childbearing age OR women or woman)) AND (knowledge OR attitudes OR beliefs OR experiences OR perceptions)) AND (Interviews OR focus groups or observations)) AND (qualitative)	All Fields	11
S1	((((perinatal cannabis use OR cannabis use during pregnancy OR cannabis use while breastfeeding OR cannabis use before pregnancy) AND (women of childbearing age OR women OR woman)) AND (view* OR experience* OR opinion OR attitude* OR perception* or belief* OR feel* OR know* OR understand*)) AND (interview OR survey OR focus group OR case stud* OR observe*)) AND (qualitative)	All Fields	11

**Table 2:** Inclusion and exclusion criteria

Criteria	Include	Exclude	Rationale
Date	2012 and after	2011 and before	Recreational cannabis was first legalized in the State of Colorado in 2012.
Topic	Research reports related to perinatal cannabis use knowledge, attitudes, beliefs, or experiences	Research reports not related	Focus is on perinatal cannabis use perceptions and experiences.
Location	Places where cannabis is legal for recreational adult use	Research reports conducted in places without legal recreational cannabis use.	Focus is on childbearing-aged women living in places recreational cannabis legalization.
Age	18–44 years	Research reports with participants under 18 and over 44 years	Population of interest are adult women of childbearing age. The Centers for Disease Control and Prevention defines the reproductive age for women as 15 to 44 years (Centers for Disease Control and Prevention, 2014).
Sex	Women only	Research reports with participants who are not women	Men who have undergone anatomical alterations by surgical intervention may have different pregnancy and birthing perceptions and experiences.
Publications	Qualitative research reports; Peer-reviewed publications	Non-qualitative research reports; Grey literature; Non-peer-reviewed publications	Conducting a theory-generating qualitative meta-synthesis of qualitative research reports that have been formally peer-reviewed.

### Search Outcomes

**Fig. 1:**
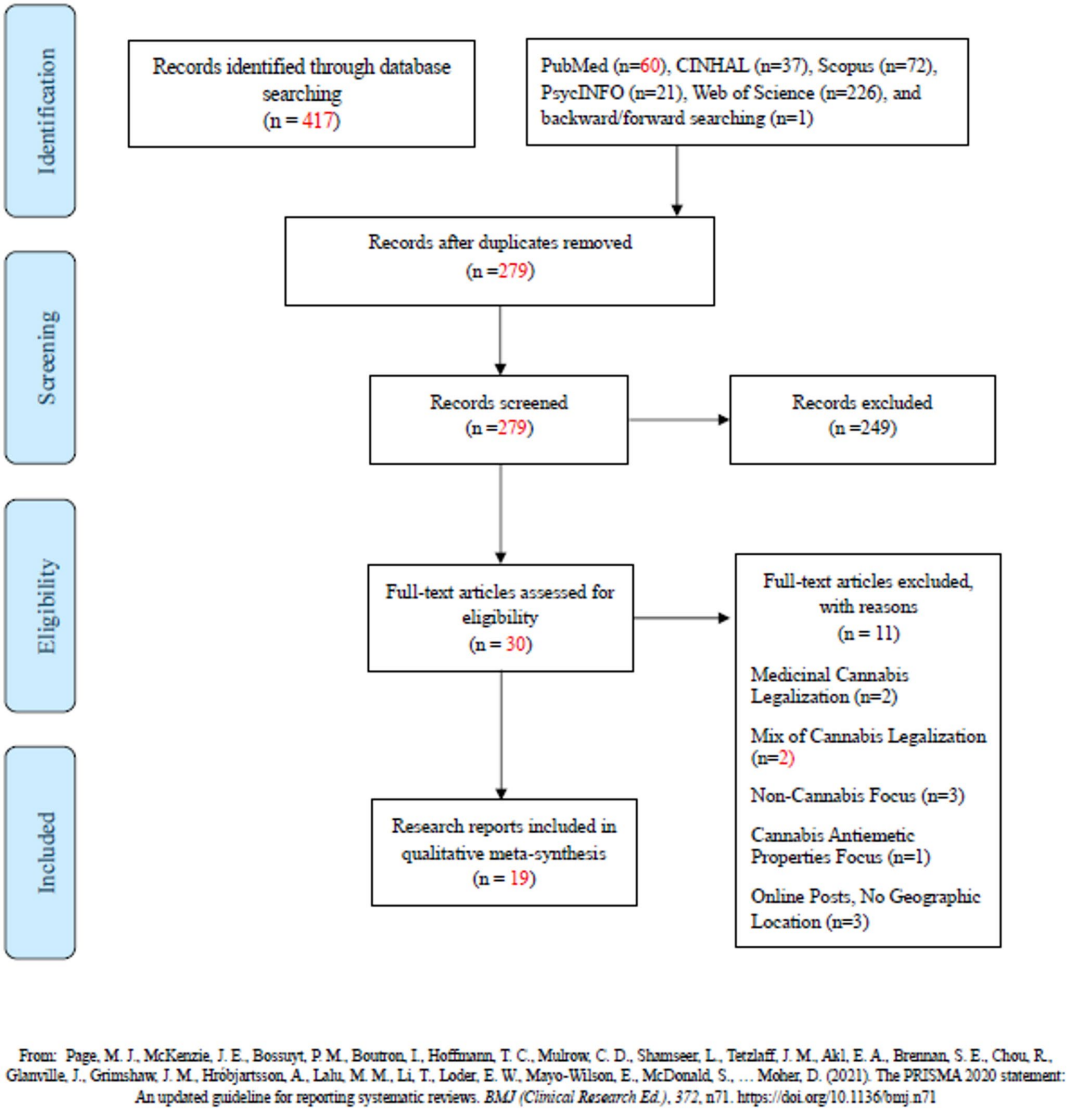
PRISMA flow diagram

Over 400 published research reports were located (Fig. 1). After removing duplicates, 279 reports were screened for eligibility by reviewing titles and abstracts. Two hundred and forty-nine reports were excluded. Full texts of the remaining 30 reports were retrieved and reviewed. Another 11 reports were excluded because they had a non-cannabis focus (*n* = 3), focused on the antiemetic properties of cannabis (*n* = 1), consisted of online posts without geographic information (*n* = 3), were conducted in US states with only medicinal cannabis legalization (*n* = 2), or included US states with mixed cannabis legalization statuses (e.g., recreational vs. medicinal) or no form of cannabis legalization at all (*n* = 2). Nineteen research reports met were included in this QMS. Of the 19 final reports, five were secondary qualitative analyses from the same author group of two primary qualitative research reports also included (Vanstone et al., 2021; Young-Wolff et al., 2022). The rationale for including these reports is that each report addressed a different research question; therefore, there was no expectation that their inclusion would inflate overall findings.

### Data Extraction and Analysis

Study characteristics were extracted to familiarize the authors with the study samples and attributes (i.e., purpose, theoretical framework, methods, etc.) (Finfgeld-Connett, 2018). This information was analyzed by summarizing attributes across the reports including article citation, geographic location, study purpose, methods, and sample characteristics (Table 3). We identified the qualitative findings from included research reports, which then served as the raw data analyzed. Raw data included the original authors’ codes, categories, and themes that were primarily found in the results section. Any qualitative findings presented in tables or as direct participant quotes were not considered raw data and were excluded (Finfgeld-Connett, 2018). Extraction of data consisted of copying the full descriptions of the data into a Microsoft Word document and importing those documents into Dedoose qualitative coding software. All extracted data were checked against the research report to prevent transcribing errors and decontextualization of the original qualitative data.

**Table 3:** CASP: quality appraisal table

Research reports	Was there a clear statement of the aims of the research?	Is a qualitative methodology appropriate?	Was the research design appropriate to address the aims of the research?	Was the recruitment strategy appropriate to the aims of the research?	Was the data collected, in a way that addressed the research issue?	Has the relationship between researcher and participants been adequately considered?	Have ethical issues been taken into consideration?	Was the data analysis sufficiently rigorous?	Is there a clear statement of findings?	How valuable is the research?
Barbosa-Leiker et al. (2020)	Y	Y	Y	Y	Y	N	Y	Y	Y	Y
English and Greyson (2022)	Y	Y	NA	Y	Y	Y	Y	Y	Y	Y
Foti et al. (2023)	Y	Y	NA	Y	Y	Y	Y	Y	CT	Y
Gould et al. (2024a, b)	Y	Y	CT	Y	Y	Y	N	Y	Y	Y
Gould et al. (2024a, b)	Y	Y	CT	Y	Y	N	N	Y	Y	Y
Greene et al. (2023)	Y	Y	Y	CT	Y	N	Y	Y	CT	Y
Kiel et al. (2023)	N	Y	NA	Y	Y	N	Y	Y	N	Y
Macario and Thomas et al. (2022)	Y	Y	NA	Y	Y	Y	Y	Y	Y	Y
McCoy et al. (2023)	Y	Y	NA	Y	Y	N	Y	Y	Y	Y
Vanstone et al. (2021)	Y	Y	Y	Y	Y	Y	Y	Y	Y	Y
Popoola et al. (2023)	Y	Y	Y	Y	Y	Y	Y	Y	Y	Y
Taneja et al. (2023)	Y	Y	NA	Y	Y	Y	Y	Y	CT	Y
Woodruff et al. (2021)	Y	Y	NA	Y	Y	CT	Y	Y	Y	Y
Young-Wolff et al. (2022)	Y	Y	NA	Y	Y	N	Y	Y	Y	Y
Young-Wolff et al. (2024a, b)	Y	Y	NA	Y	Y	Y	Y	Y	Y	Y
Mian et al. (2023)	Y	Y	NA	Y	Y	N	Y	Y	Y	Y
Barbosa-Leiker et al. (2025)	Y	Y	Y	Y	Y	Y	Y	Y	Y	Y
Denson et al. (2025)	Y	Y	NA	Y	Y	CT	Y	Y	Y	Y
Odgen et al. (2025)	Y	Y	NA	Y	Y	Y	Y	Y	Y	Y

Y = ‘Yes’; N = ‘No’; CT = ‘Can’t Tell’; NA = ‘Not Applicable’

### Rigor

The purpose of theory-generating QMS research is to develop a new theory without any preexisting assumptions about coding and categorizing structures (Finfgeld-Connett, 2018). The qualitative data extracted from each research report was coded line by line and inductively organized by creating preliminary codes that captured the meaning of the findings. If a single line of data was associated with multiple codes, we examined whether they had captured multiple findings that needed to be separated and analyzed individually. Memoing was used to make sense of concepts and their dynamic relationships within and across research reports, allowing for a robust data analysis process (Finfgeld-Connett, 2018). Reflexivity was also used to ensure that personal biases were not influencing the data analysis process and to evaluate codes and memos to ensure an accurate representation of the data. The trustworthiness and credibility of the resultant theoretical framework were safeguarded through multiple strategies that were used to enhance validity, including unbiased data collection and sampling, memoing, discussions during team meetings, and reflexivity (Finfgeld-Connett, 2018).

### Quality Appraisal

The Critical Appraisal Skills Program (CASP) checklist was used to assess the quality of each research report (Table 3). CASP is the most widely used tool for appraising the quality of qualitative research reports and is endorsed by the Cochrane Qualitative and Implementation Methods Group and World Health Organization (Long et al., 2020). This 10-item tool can assess the strengths and limitations of any qualitative research method and is generally believed to be user-friendly for novice researchers.

## Results

### Attributes of Included Research Reports

All data that was included was collected following recreational cannabis legalization in the respective location. Table 4 presents the attributes of the 19 included research reports, yielding an overall sample of 397 women. Sample sizes from secondary research reports Vanstone et al. (2021) and Young-Wolff et al. (2022) were not added to the total count because the samples of their respective primary study were already included. Most of the research reports (*n* = 15) were conducted in the US and recruited participants from California (*n* = 8), Washington (*n* = 3), Colorado (*n* = 1), Massachusetts (*n* = 1), Illinois (*n* = 1), and from a combination of California, Oregon, and Washington (*n* = 1). The other four research reports were conducted in Canada where recreational cannabis use is legal nationwide. Data collection methods mainly used individual interviews (*n* = 12) and focus groups (*n* = 5). One author group used a photovoice workshop (Greene et al., 2023) and another used an online bulletin board in addition to a focus group (Macario & Thomas, 2022). Most research reports described conducting a thematic (*n* = 11) or content (*n* = 4) analysis, while the remaining four did not describe data analysis methods.

**Table 4:** Characteristics of research reports

First author & year	Geographic location	Study purpose	Methods	Sample characteristics
Barbosa-Leiker at al. (2020)	United States, Washington	To explore pregnant and postpartum women’s perceptions on the benefits and risks of using cannabis perinatally	Interviews, Content Analysis	*N* = 19 (14 pregnant; 5 postpartum), mostly white between 18 and 29 years, all self-reported cannabis use during pregnancy
English and Greyson (2022)	United States, Massachusetts	To explore how cannabis legalization effects pregnant and breastfeeding women’s perceptions and decision-making about using cannabis perinatally	Semi-structured interviews by phone, Content Analysis	*N* = 23, 52% 20–29 years, 74% were prior cannabis users and also used during pregnancy and/or breastfeeding
Gould et al. (2024a)	United States, California	To explore pregnant women’s perceptions and patterns of cannabis use, including influences of socio-environmental factors	Semi-structured virtual interviews, Thematic Analysis	*N* = 19, all over 21 years, self-identified as BIPOC (Black, Indigenous, People of Color), and self-reported cannabis use during pregnancy
Gould et al. (2024b)	United States, California	To explore perceptions about cannabis use during pregnancy among maternal healthcare providers and pregnant cannabis using patients	Semi-structured virtual interviews	*N* = 7, 57% Hispanic, Mean age = 27 years; Providers: 70% white, 60% 30–59 years
Greene et al. (2023)	Canada	To explore cannabis-using pregnant and breastfeeding women’s experiences with cannabis use stigma and surveillance by health and social care providers	Virtual photovoice workshops, Thematic Analysis	*N* = 23, 57% Black or Indigenous, all self-reported using cannabis perinatally
Kiel et al. (2023)	United States, Washington	To explore postpartum women’s beliefs, decision-making, and experiences with perinatal cannabis use	Semi-structured interviews	*N* = 15, 67% white, 73% self-reported continued cannabis use during pregnancy
Macario and Thomas (2022)	United States, Washington	To explore pregnant/breastfeeding and non-pregnant/breastfeeding childbearing aged women’s attitudes and beliefs about perinatal cannabis use	Virtual focus groups and online bulletin board discussions, Thematic Analysis	*N* = 95 (67 current cannabis users; 28 non-cannabis users), 66% white, Mean age = 32 years
McCoy et al. (2023)	United States, Colorado	To explore rural pregnant and non-pregnant childbearing aged women’s perceptions and experiences related to maternal cannabis use	Semi-structured interviews, Thematic Analysis	*N* = 9, demographics unavailable
Vanstone et al. (2021)	Canada	To explore why pregnant and breastfeeding women’s use cannabis and changes in motivations across perinatal periods	Semi-structured virtual or phone interviews	*N* = 52 (30 pregnant; 22 breastfeeding), 69% white, 75% 19–34 years, all self-reported using cannabis before pregnancy
Popoola et al. (2023) *	Canada	To explore pregnant and breastfeeding women’s perceptions of perinatal cannabis use risks and mitigation strategies	Semi-structured virtual or phone interviews, Content Analysis	*N* = 52 (30 pregnant; 22 breastfeeding), 69% white, 75% 19–34 years, all self-reported using cannabis before pregnancy
Taneja et al. (2023) *	Canada	To explore pregnant and breastfeeding women’s information-seeking behaviors about perinatal cannabis use	Semi-structured virtual interviews, Content Analysis	*N* = 52 (30 pregnant; 22 breastfeeding), 69% white, 75% 19–34 years, all self-reported using cannabis before pregnancy
Woodruff et al. (2021)	United States, California	To explore pregnant and postpartum women’s experiences about discussing their cannabis use with healthcare providers	Semi-structured interviews, Thematic Analysis	*N* = 33, 50% Black or Hispanic, mean age = 29 years, all self-reported using cannabis at least weekly before pregnancy
Young-Wolff et al. (2022)	United States, California	To explore pregnant women’s perceptions about recreational cannabis legalization and cannabis use behaviors	Semi-structured virtual focus groups, Thematic Analysis	*N* = 53, 57% white, mean age = 30 years, 70% self-reported using cannabis daily during pregnancy
Young-Wolff et al. (2024b) *	United States, California	To examine cannabis-using pregnant women’s opinions and experiences regarding their intentions to use cannabis in the postpartum period	Semi-structured virtual focus groups, Thematic Analysis	*N* = 53, 57% white, mean age = 30 years, 70% self-reported using cannabis daily during pregnancy
Mian et al. (2023) *	United States, California	To examine pregnant women’s perceptions and patterns of cannabis use, including modes of administration	Semi-structured virtual focus groups, Thematic Analysis	*N* = 53, 57% white, mean age = 30 years, 70% self-reported using cannabis daily during pregnancy
Foti et al. (2023) *	United States, California	To explore pregnant women’s perceptions of cannabis use during pregnancy and healthcare experiences	Semi-structured virtual focus groups, Thematic Analysis	*N* = 53, 57% white, mean age = 30 years, 70% self-reported using cannabis daily during pregnancy
Barbosa-Leiker et al. (2025)	United States, California, Oregon, Washinton	To explore pregnant and postpartum American Indian women’s perceptions of risks and benefits of perinatal cannabis use	Semi-structured virtual or in-person interviews, Thematic Analysis	*N* = 10 (5 pregnant; 5 postpartum), 100% American Indian, mean age = 29 years, 90% self-reported using cannabis regularly during pregnancy and 10% only during postpartum
Denson et al. (2025)	United States, illinois	To examine postpartum and future pregnant women’s beliefs and perceptions of perinatal cannabis use	Semi-structured virtual interviews or focus groups, Thematic Analysis	*N* = 20, 50% Black or African American, 50% White, 10% Hispanic or Latina, mean age = 30 years, 4.4% self-reported current daily or weekly use of cannabis
Odgen et al. (2025)	United States, California	To explore postpartum women’s motivations for perinatal cannabis use and desire for interventions	Semi-structured interviews, Thematic analysis	*N* = 17, 53% White, 23.5% Black, 23.5% Hispanic, mean age = 26 years, 71% self-reported daily cannabis use during postpartum and 59% during breastfeeding

*Secondary analysis

### Identified Themes

Eight primary themes impacting decision-making about perinatal cannabis use were identified and are detailed below.

### Recreational Cannabis Legalization

The nuances surrounding recreational cannabis legalization, such as the applicability of the policy and legal implications for this population, resulted in both uncertainty and acceptance of perinatal cannabis use. Several women discussed disparities in public health messaging about the policy of legal recreational cannabis use for those who are pregnant or breastfeeding (Barbosa-Leiker et al., 2020; English & Greyson, 2022; McCoy et al., 2023). For example, women expressed concerns about whether the legal status of cannabis was similar to that of alcohol and tobacco or if it was still considered an illicit substance because it remains illegal federally (English & Greyson, 2022). Conversely, some women were more open to discussing their perinatal cannabis use following legalization, under the misconception that it was an indicator that cannabis was permitted and safe to consume (Young-Wolff et al., 2022). Cannabis retailers (e.g., cannabis dispensaries) and budtenders (i.e., people who work at dispensaries) were frequently relied on as a source for cannabis information and products (Mian et al., 2023; Young-Wolff et al., 2022). Women believed cannabis products from retailers were better regulated (i.e., safer) and that budtenders were knowledgeable (i.e., trained) (Barbosa-Leiker et al., 2020; Gould et al., 2024a; Macario & Thomas, 2022).

### Clinical Policies and Practices

Women expressed concerns about routine drug testing policies and the possibility of their infant being tested at birth (English & Greyson, 2022; Greene et al., 2023; Woodruff et al., 2021). Most women shared fears of being reported to child protection services (CPS) if their perinatal cannabis use was discovered (Barbosa-Leiker et al., 2020; English & Greyson, 2022; Foti et al., 2023; Greene et al., 2023; Macario & Thomas, 2022; Popoola et al., 2023; Woodruff et al., 2021; Young-Wolff et al., 2022). Women believed CPS involvement would increase their risk of surveillance or having their infant removed from the home (English & Greyson, 2022; Macario & Thomas, 2022; Woodruff et al., 2021). Low-income and racial and ethnic minority women were particularly concerned about legal repercussions, with many indicating that their identity put them at higher risk for drug testing and CPS involvement (Foti et al., 2023; Greene et al., 2023).

### Stigma and Discrimination

Many women perceived various sources and forms of stigma (e.g., health care clinicians, social, and anticipated stigma) regarding their perinatal cannabis use. Stigma from clinicians was commonly reported, and women believed that recommendations were based on the clinicians’ personal biases rather than medical expertise. Some women suggested up-to-date knowledge and training about perinatal cannabis use are needed to destigmatize discussions and promote patient-centered care (English & Greyson, 2022; McCoy et al., 2023). Anticipated stigma or preconceived notions also affected women’s decisions and limited their disclosure of perinatal cannabis use (Foti et al., 2023; Gould et al., 2024a; Odgen et al., 2025; Vanstone et al., 2021). Some women from historically marginalized populations reported discrimination by clinicians and social workers, refraining them from inquiring for information and increasing punitive fears (English & Greyson, 2022; Foti et al., 2023; Greene et al., 2023).

### Access and Desire for Information

Most women emphasized the importance and need for evidence-based information about the safety, risks, and effects of using cannabis during the perinatal period (Denson et al., 2025; Popoola et al., 2023; Taneja et al., 2023; Young-Wolff et al., 2024b). Women expressed that current scientific research and medical information provided little guidance about the safety of perinatal cannabis use and lacked conclusive results on its effects (Denson et al., 2025; Foti et al., 2023; Macario & Thomas, 2022; McCoy et al., 2023). This inconsistency contributed to the predominant concern of being misinformed (Greene et al., 2023). As a result, women searched for their own information online or asked others about their beliefs and experiences with perinatal cannabis use (English & Greyson, 2022; Foti et al., 2023; Gould et al., 2024a; Kiel et al., 2023; Macario & Thomas, 2022; McCoy et al., 2023; Odgen et al., 2025; Taneja et al., 2023; Woodruff et al., 2021).

### Clinician-Patient Relationships

Clinicians provided varied levels of perinatal health care and support to their patients. Most women experienced feelings of distrust and were uncomfortable bringing up perinatal cannabis use with their clinician (English & Greyson, 2022; Greene et al., 2023; McCoy et al., 2023; Woodruff et al., 2021). Some women reported wanting to engage in open conversations about their cannabis use and reasons for consumption, but only a few did (English & Greyson, 2022; Foti et al., 2023; Odgen et al., 2025; Taneja et al., 2023; Woodruff et al., 2021). Women engaging in conversations with clinicians noticed the focus often shifted from their health as pregnant/parenting patients toward educating about the negative effects on the developing fetus or infant, leading them to feel unheard and uncared for (Barbosa-Leiker et al., 2020; Gould et al., 2024b; Young-Wolff et al., 2024b). Clinicians generally encouraged abstinence over harm reduction approaches designed to reduce negative health outcomes (Barbosa-Leiker et al., 2020, 2025; Denson et al., 2025; Greene et al., 2023). Women received mixed messages and insufficient information about perinatal cannabis use, leading some to question their clinician’s medical expertise (English & Greyson, 2022; Kiel et al., 2023; Odgen et al., 2025; Taneja et al., 2023; Woodruff et al., 2021). Others experienced a complete lack of communication, with several women claiming that their clinicians did not ask them about or follow up on their perinatal cannabis use at all (Barbosa-Leiker et al., 2025; Gould et al., 2024b; Woodruff et al., 2021). Women from historically marginalized populations expressed a significant lack of support and cultural incompetence from clinicians altogether, creating a desire for services from people with similar backgrounds (Foti et al., 2023; Greene et al., 2023; Odgen et al., 2025).

### Most Trusted Sources: Family and Friends

Women frequently turned to family and friends to ask for information about their personal experiences with perinatal cannabis use (Denson et al., 2025; Foti et al., 2023; Macario & Thomas, 2022; Taneja et al., 2023; Woodruff et al., 2021). Women made decisions based on these personal narratives because they were valid firsthand experiences from people who had been in the same situation (Denson et al., 2025; Mian et al., 2023; Taneja et al., 2023). Some described valuing their partners’ input and support in their decisions to use cannabis perinatally (Kiel et al., 2023; Mian et al., 2023; Popoola et al., 2023). Other women concealed their use from friends or family out of fear of stigma or judgment (English & Greyson, 2022; Taneja et al., 2023).

### Perceptions and Experiences

Decisions were primarily influenced by their perceptions of the benefits and risks of cannabis, as well as the potential effects on fetal or infant health. The subthemes below provide a full description of the key factors driving decision-making.

#### Cannabis Benefits and Risks

Many women weighed the risks and benefits of perinatal cannabis use, particularly as they sought to mitigate their own needs against potential harm to their fetus/infant. They generally perceived cannabis as a ‘natural’ medicine or plant that was more effective in treating their symptoms compared to prescription medications (Foti et al., 2023; Greene et al., 2023; Kiel et al., 2023; Macario & Thomas, 2022; Odgen et al., 2025; Vanstone et al., 2021). Cannabis was perceived as a ‘safer’ alternative with fewer harmful risks for their fetus/infant, and for themselves. Some women drew comparisons between cannabis and other substances with well-established risks (e.g., alcohol, tobacco, opioids, etc.) to justify their beliefs and decisions to use cannabis perinatally (Barbosa-Leiker et al., 2020, 2025; Denson et al., 2025; Foti et al., 2023; Greene et al., 2023; Kiel et al., 2023; Macario & Thomas, 2022; McCoy et al., 2023; Young-Wolff et al., 2024b). Other women shared using cannabis as a harm reduction strategy or substitute to decrease their use of higher-risk substances, such as methamphetamine, heroin, or opioids (Barbosa-Leiker et al., 2025; Greene et al., 2023; Kiel et al., 2023). Ultimately, most believed that the benefits of cannabis for managing their symptoms outweighed the known harmful risks to their fetus/infant.

#### Impact on Fetal/Infant Health

Primary concerns about using perinatal cannabis included the effects that it could have on fetal/infant health (Barbosa-Leiker et al., 2020; Denson et al., 2025; Gould et al., 2024b; Kiel et al., 2023; McCoy et al., 2023; Mian et al., 2023; Popoola et al., 2023; Taneja et al., 2023; Vanstone et al., 2021; Young-Wolff et al., 2024b). Women observed the impact of their cannabis use during their pregnancy or after giving birth by looking for specific symptoms or signs of harm. They perceived miscarriage, premature delivery, LBW, asthma, ADHD, and neurodevelopmental issues as risks of perinatal cannabis use (Kiel et al., 2023; Popoola et al., 2023). To these women, the absence of these adverse outcomes indicated that their infant was not affected by their use. Others turned to the well-established harmful effects of tobacco use during pregnancy on fetal development to understand the potential risks of smoking cannabis (Mian et al., 2023). Several women also perceived that cannabis use during breastfeeding was more dangerous than cannabis use during pregnancy because they believed THC could more directly reach the infant through breast milk (Barbosa-Leiker et al., 2020; Kiel et al., 2023; Young-Wolff et al., 2024b). However, there were also mixed perceptions about the effects of consuming cannabis during breastfeeding (Barbosa-Leiker et al., 2025; Odgen et al., 2025).

#### Managing Consumption and Safety

Due to the possible risks associated with cannabis on fetal/infant health, many women changed their cannabis use during the perinatal period as a precautionary measure (Barbosa-Leiker et al., 2025; Foti et al., 2023; Kiel et al., 2023; Popoola et al., 2023; Taneja et al., 2023; Vanstone et al., 2021; Woodruff et al., 2021; Young-Wolff et al., 2022; Young-Wolff, Green, Young-Wolff et al., 2024b). While some described ceasing their cannabis use upon learning about their pregnancy, others reduced their frequency to mitigate risks (Foti et al., 2023; Popoola et al., 2023; Vanstone et al., 2021; Woodruff et al., 2021; Young-Wolff, Green, Young-Wolff et al., 2024b). Women who stopped during pregnancy typically planned to resume use after birth or breastfeeding (Odgen et al., 2025; Popoola et al., 2023; Vanstone et al., 2021; Young-Wolff, Green, Young-Wolff et al., 2024b). Most who continued to use cannabis throughout pregnancy or breastfeeding changed their mode of consumption, such as from inhalation to edible products, opted for using tobacco-free papers to smoke cannabis, or managed levels of THC (Barbosa-Leiker et al., 2025; Gould et al., 2024b; McCoy et al., 2023; Mian et al., 2023; Popoola et al., 2023). They also expressed preferences on purchasing cannabis, with many turning to dispensaries they trusted or family members who grew their own cannabis (Gould et al., 2024b; Mian et al., 2023; Young-Wolff et al., 2022). Breastfeeding women described switching to formula feeding or ‘pumping and dumping’ breast milk (i.e., pumping breast milk after consuming cannabis and throwing it away instead of feeding it to their infant) to continue using cannabis (Kiel et al., 2023; Young-Wolff, Green, Young-Wolff et al., 2024b). Other breastfeeding women increased their hygiene (i.e., washing hands or body and changing their clothes before coming into contact with their infant, smoking cannabis outside or at work instead of at home, etc.) or managing time between cannabis use and breastfeeding (i.e., using cannabis well before or shortly after breastfeeding) (Odgen et al., 2025; Popoola et al., 2023; Young-Wolff, Green, Young-Wolff et al., 2024b).

### Self-management

Most women described using cannabis to self-medicate perinatal mental health symptoms including stress, anxiety, trauma, and depression (Barbosa-Leiker et al., 2020, 2025; Denson et al., 2025; English & Greyson, 2022; Foti et al., 2023; Gould et al., 2024a; Gould, Ganesh, Nguyen, Gould et al., 2024a, b; Greene et al., 2023; Kiel et al., 2023; Macario & Thomas, 2022; Mian et al., 2023; Odgen et al., 2025; Vanstone et al., 2021; Woodruff et al., 2021; Young-Wolff et al., 2024). Cannabis was also used to self-manage pregnancy-related symptoms, such as nausea and vomiting, loss of appetite, pain, weight gain, and difficulty sleeping (Barbosa-Leiker et al., 2025; Denson et al., 2025; Foti et al., 2023; Macario & Thomas, 2022; Mian et al., 2023; Vanstone et al., 2021; Woodruff et al., 2021), as well as symptoms from chronic conditions, like fibromyalgia or chronic pain (Kiel et al., 2023; Macario & Thomas, 2022). While most continued to use cannabis for its symptom management benefits, others discontinued use due to concerns about the effects on fetal/infant health (Foti et al., 2023; Popoola et al., 2023; Vanstone et al., 2021; Young-Wolff et al., 2022). However, some women experienced negative effects and believed cessation worsened their symptoms, resulting in cannabis reuptake (English & Greyson, 2022). Women who previously used cannabis perinatally and whose children had not developed any health problems felt more comfortable using it again (Kiel et al., 2023; Macario & Thomas, 2022; Popoola et al., 2023; Young-Wolff et al., 2022). Concurrently, those who experienced pregnancy or birth complications questioned whether this was a result of their perinatal cannabis use (Kiel et al., 2023; Popoola et al., 2023).

### Perinatal Cannabis Use Decision-Making Model

**Fig. 2:**
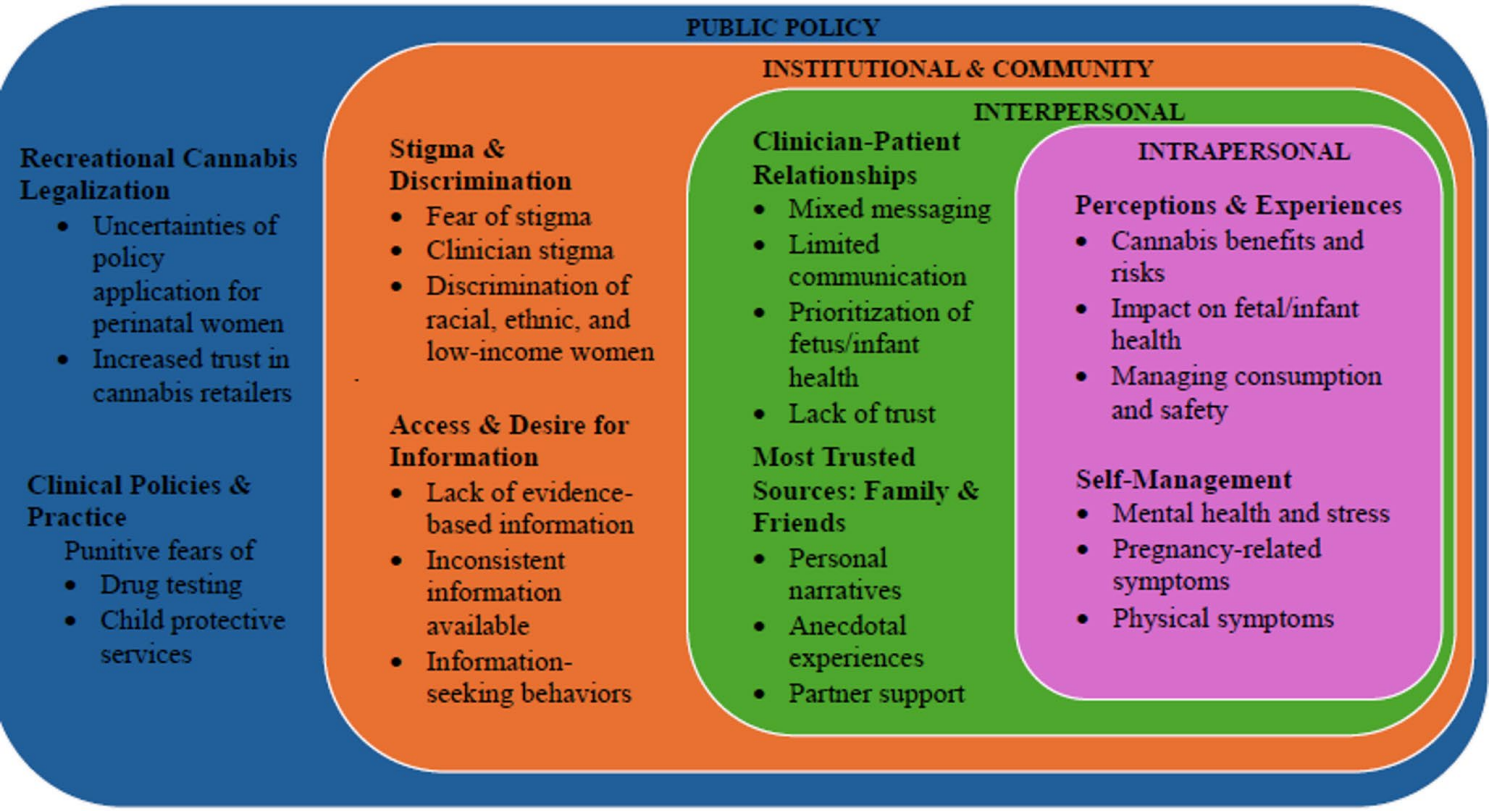
Perinatal cannabis use decision-making model

Our findings resulted in eight primary themes and subthemes that served as constructs of the perinatal cannabis use decision-making model (Fig. 2). Based on Bronfenbrenner at al.‘s (1977) original Socio-Ecological Model, our model emphasizes the interdependencies of critical public policy, institutional and community, interpersonal, and intrapersonal factors that influence women’s decision-making. Specifically, our model identifies factors that require both public health (e.g., clear cannabis messaging for pregnant/parenting women, perinatal cannabis use education, etc.) and clinical interventions (e.g., non-stigmatizing and patient-centered care conversations, evidence-based and up-to-date information, etc.) to help women make better, more informed decisions when considering using cannabis perinatally.

## Discussion

Legalization implications have led to an increased perception of safety due to its regulation, resulting in a potential higher likelihood of perinatal cannabis use. Our findings support quantitative studies demonstrating that women are more likely to use cannabis during the perinatal period in US states where cannabis is legal recreationally compared to US states where cannabis is not legal recreationally (Skelton et al., 2020; Sood et al., 2022; Vachhani et al., 2022). The reliance on cannabis retailers for products and information is concerning because there is no consistent legislation for licensing across states that legalized recreational cannabis use, resulting in both licensed and unlicensed facilities (Unger et al., 2020). There is also contradicting evidence that dispensaries are counseling pregnant women about using cannabis to treat pregnancy-related symptoms, such as nausea and vomiting, in places with legalized recreational use (Dickson et al., 2018; Vastis et al., 2021). We acknowledge that recreational legalization is for the general adult population and that legalizing cannabis has broader benefits, including for societal (e.g., justice decriminalization, taxation, and product regulation) and therapeutic purposes (e.g., anti-emesis, pain relief, appetite stimulation, etc.).

On the other hand, limited studies have examined attitudes toward cannabis during pregnancy in places where recreational use is not legalized. For example, Ramseyer et al.’s (2024) cross-sectional survey study indicates pregnant cannabis users and nonusers’ share permissive legalization attitudes, minimal perceived risks, lack of counseling, and desire for more information about risks. Other survey-based studies suggest women’s mixed attitudes toward legalization (Mark et al., 2017) and beliefs of slight to no risk of harm (Ko et al., 2015). Moreover, Keyhani et al.’s (2018) survey study indicates that the general US adult population believed cannabis was beneficial for managing pain, anxiety, stress, and depression. Although these attitudes emerged in settings without recreational legalization, their findings closely reflect the themes identified in our qualitative meta-synthesis, suggesting broader societal or cultural patterns. Regardless of legalization, policymakers, public health, and clinicians should provide clearer communications about perinatal cannabis use and its potential medical or legal consequences to better support informed decision-making among pregnant and parenting women.

A strong need for evidence-based information about the safety, risks, and effects of perinatal cannabis use was emphasized by women in our study, similar to prior research (Jarlenski et al., 2016; Jarlenski & Spencer, 2022). Most information available was perceived as unclear and inconsistent, which did not help guide decision-making about perinatal cannabis use. This resulted in the same information-seeking behaviors (i.e., online searching and reliance on family and friends’ personal experiences) that women reported in Jarlenski et al.‘s (2016) study nearly a decade ago, before medicinal cannabis legalization. Consistent with recent studies, women described turning to online posts on forums, discussion boards, or social media (Lebron et al., 2022; Micalizzi et al., 2024; Oram et al., 2018). Our study aligns with Chang et al.‘s (2019) findings suggesting that women base their perinatal cannabis use decisions on their knowledge about other substances (e.g., tobacco and alcohol) due to the lack of information about the effects of cannabis.

Most importantly, our findings raised the issue of how pregnant and parenting women perceive cannabis use risks in the absence of negative effects or even when effects are present, they doubt it is due to their use. Based on limited research (Goodin et al., 2025; McKenzie et al., 2022; Satti et al., 2022;) and our own findings, we speculate the benefits gained from using cannabis perinatally at that point in time are driving factors shaping risk perceptions and continued use. This may be particularly relevant for women who are experiencing higher volumes of stress (Satti et al., 2022), pregnancy-related symptoms (McKenzie et al., 2022), and uncertainty about the risks of using cannabis during the perinatal period (Goodin et al., 2025). As such, our findings warrant further investigation and the development of public health campaigns and evidence-based educational materials to clearly inform women about perinatal cannabis use effects, risks, and safety.

Clinicians play a critical role in perinatal cannabis use decision-making, specifically when providing care and delivering health messages to their pregnant or parenting patients. Our findings indicate that clinician’s engagement in delivering perinatal health care and discussing cannabis use varied substantially, similar to other studies (Bayrampour et al., 2019). Most women didn’t disclose their cannabis use or engage in conversations with clinicians due to fears of being reported to CPS, supporting Jarlenski and colleagues (2016) findings. Additionally, limited or unclear communications decreased women’s trust in clinicians and generated feelings of being unheard as patients, similar to women who misused opioids perinatally (Morton et al., 2023). Others reported not receiving any type of care at all, as highlighted by other women in Bayrampour et al.‘s (2019) review. Further, an ethical issue raised in some of our findings is the notion that cannabis may be safer than other substances, especially for people who have a history of using higher-risk substances (e.g., methamphetamine, heroin, opioids) (Barbosa-Leiker et al., 2025; Greene et al., 2023; Kiel et al., 2023). Future work could focus on examining whether cannabis is a better alternative for people who use higher-risk substances, particularly during the perinatal period.

There are social issues to consider. Several women described experiencing stigma and discrimination from clinicians for their cannabis use during pregnancy or breastfeeding, like a review examining the effects of stigma on substance-using pregnant people to improve perinatal care (Weber et al., 2021). Additionally, discrimination and inadequate culturally competent care were highlighted by women from historically marginalized populations, consistent with Murphy et al.’s (2022) review exploring perinatal experiences among Black women. These experiences can inflict a significant lack of trust in clinicians, resulting in women searching for information on their own, relying on friends and family, and self-medication practices. Consistent with other quantitative studies, our findings capture an uptake in self-medication practices to manage mental health and pregnancy-related symptoms (Vanstone et al., 2022). Now is a seemingly pivotal time for clinicians, including nurses, to focus on building trusted relationships with women to screen for and discuss perinatal cannabis use in a non-stigmatizing and culturally competent manner. Nurse-patient communication interactions are well-documented and can be leveraged to improve perinatal and cannabis healthcare delivery (Höglander et al., 2023).

### Limitations

Relevant research reports may have been overlooked due to limitations in the search terms, databases, and inclusion and exclusion criteria used. Five research reports conducted secondary qualitative analyses of primary research reports were included, potentially skewing results because they used the same sample. We focused on locations where recreational cannabis use is legalized and on women of childbearing age living there, potentially limiting the applicability of these findings to different populations and settings. Finally, the sensitive and frequently stigmatized nature of perinatal cannabis use may have influenced participant responses, limiting the richness of qualitative findings we analyzed. These limitations should be considered when interpreting our study’s findings and implications.

## Conclusion

Our QMS highlights the complex interactions of social and contextual factors that influence women’s perceptions and experiences with perinatal cannabis use. Our findings provide insight into decision-making about whether to use cannabis during the perinatal period and are consistent with available empirical evidence. Through targeted public health interventions and clinical support strategies, we can move closer to addressing the complexities of cannabis use during the perinatal period, especially in a time of expanding recreational cannabis legalization and social acceptance.

## Data Availability

Available upon request to authors.

## Code Availability

Available upon request to authors.
